# Integrated analysis of autophagy and ferroptosis mediating aristolochic acid A-induced nephrotoxicity via transcriptome and m6A epigenetic transcriptome in HK-2 cells

**DOI:** 10.3389/fgene.2026.1873549

**Published:** 2026-09-23

**Authors:** Peichun Ye, Wenqiang Bao, Dongcheng Zhang, Jiaxin Wen, Wei Zhao, Shuqi Huang, Lele Yang, Hong Hu, An Zhu

**Affiliations:** 1 Key Laboratory of Gastrointestinal Cancer (Fujian Medical University), Ministry of Education, Fuzhou, China; 2 Fujian Provincial Key Laboratory of Environment and Health, Fujian Medical University, Fuzhou, China

**Keywords:** aristolochic acid A, autophagy, ferroptosis, m6A modification, oxidative stress

## Abstract

**Introduction:**

Aristolochic acid A possesses anti-inflammatory, antispasmodic and diuretic pharmacological activities, while exerting strong toxic effects that cause irreversible renal tubular injury, renal fibrosis and malignant tumors such as urothelial carcinoma. This study aimed to elucidate the relationship between m6A methylation and AAA-induced nephrotoxicity.

**Methods:**

HK-2 cells were treated with AAA. Cell viability was detected to evaluate AAA-induced cytotoxicity. mRNA-seq and MeRIP-seq were performed to screen differentially expressed genes and identify m6A methylation profiles related to renal injury. Combined bioinformatics analysis and cellular validation experiments were used to explore the key regulatory factor and related pathological mechanisms.

**Results:**

AAA significantly reduced the viability of HK-2 cells. mRNA-seq identified differentially expressed genes closely associated with oxidative stress and autophagy under AAA treatment. MeRIP-seq confirmed that m6A methylation alterations were tightly correlated with the expression of oxidative stress, autophagy and ferroptosis-related genes. IGF2BP2 was identified as a key mediator of AAA-induced cytotoxicity. Functional experiments further verified that AAA triggered oxidative stress, autophagy and ferroptosis in HK-2 cells, in which IGF2BP2 exerted a critical regulatory role.

**Discussion:**

These findings demonstrate that AAA-induced renal tubular cell damage is closely related to altered m6A methylation levels and dysregulated expression of m6A regulatory factors in target genes, revealing a novel epigenetic mechanism underlying AAA nephrotoxicity.

## Highlights

- Combined transcriptome and m6A epitranscriptome sequencing was employed.
- IGF2BP2 was implicated as a potential mediator of the cytotoxicity induced by AAA in HK-2 cells.
- The crosstalk among oxidative stress, autophagy and ferroptosis was elucidated to jointly mediate AAA-induced nephrotoxicity.

## 1 Introduction

Aristolochic acid A (AAA) is a nitrophenanthrene carboxylic acid derived mainly from herbal plants, including Asarum and Aristolochia (Gong et al., 2026). AAA-containing drugs have been used for decades to treat a variety of conditions such as urinary tract infections, hepatitis, edema and eczema-among others (Anger et al., 2020; Xie et al., 2024).

The toxicity of AAA is mainly reflected in renal toxicity and carcinogenicity, and the core is the damage of its metabolites to cells. After AAA enters human body, it is metabolized by liver to produce aristoloactam with strong oxidation. This metabolite will combine with DNA and protein in renal tubular epithelial cells, destroy cell structure and function, and lead to renal tubular necrosis. Long-term function can also cause renal interstitial fibrosis, which eventually leads to irreversible chronic renal failure, named aristolochic acid nephropathy (Lukinich-Gruia et al., 2022; Kwok et al., 2025; Kocic et al., 2021). Aristrolochic acid A and its active metabolite Aristolactam I both disrupted circadian rhythms in U2OS human osteosarcoma cells and mRTEC mouse renal tubular epithelial cells, highlighting the damaging effects of aristolochic acids on the kidney’s internal clock and the resulting aggravation of renal injury (Xie et al., 2024). AAA was first described as a potent human carcinogenic and renal toxic compound in the early 1990s, when hundreds of cases of kidney failure and urothelial tumors were reported in Belgium and other Western European countries from eating Chinese slimming pills contaminated with AAA (Vanherweghem et al., 1993). Aristolactam can form stable DNA adducts with cell DNA, which will interfere with DNA replication and repair, resulting in gene mutation, especially genes related to urinary system tumors, and long-term accumulation will induce malignant tumors such as bladder cancer and kidney cancer (Das et al., 2022; Arlt et al., 2002). Moreover, once this DNA adduct is formed, it is difficult to be removed by the body, and its toxicity has accumulation. The International Agency for Research on Cancer has categorized this compound as a Group 1 carcinogen.

Apparent modifications are heritable chemical modifications that do not change DNA nucleotide sequences and affect gene expression activity by regulating the structure and function of nucleic acids or histones. They can be divided into three categories, DNA modification, Histone modification and RNA modification. m6A is a typical type of RNA modification (Huang et al., 2024).

Early research has demonstrated a potential link between m6A and the toxicological effects of traditional herbal medicines (Lin et al., 2024b; Wu et al., 2024; Lin et al., 2024a). Over the past few decades, approximately 160 distinct RNA modifications have been identified. These modifications encompass N4-acetylcytidine, 1-methyladenosine (m1A), 5-methylcytidine (m5C), N7-methylguanosine (m7G), N6-methyladenosine (m6A) (Huang et al., 2024), and pseudouridine. m6A is the most abundant and widely distributed internal chemical modification within eukaryotic messenger RNA. Among which m6A has the highest abundance, mainly in the termination codon of mRNA and RRACH sequence of 3′UTR. This modification is reversibly regulated: the writer complex composed of METTL3, METTL14 and WTAP mediates its installation, while reader proteins represented by YTHDF and IGFBP recognize this modification to modulate RNA splicing, translation, localization and stability. Additionally, eraser enzymes including ALKBH5 and FTO are responsible for its removal, thereby governing multiple biological processes, including cell proliferation, differentiation, metabolism and immune homeostasis (Wei, 2024). Since RNA modification was revealed in 1970, MeRIP-seq and other high-throughput technologies have finally enabled the whole picture and functional mechanism of m6A to be systematically analyzed, which has become the core hot spot of apparent transcriptomics (Meyer et al., 2012).

m6A has been shown to play critical roles in oxidative stress, autophagy, and ferroptosis, with potential crosstalk to the biological effects of AAA. Simultaneously, m6A critically regulated the response to oxidative stress. For example, the knockdown METTL3 mediated destabilization of HO-1 and GCLC mRNAs increased reactive oxygen species accumulation to aggravate oxidative stress (Li et al., 2023a). By demethylating ATG5 and ATG7 mRNAs, FTO increases their stability and drives autophagosome formation, which in turn enhances autophagy flux, a process where m6A exerts tight regulation over core effectors ATG5 and LC3 to mediate organelle elimination (Wang et al., 2020). m6A tightly regulates ferroptosis, an essential iron-dependent programmed cell death process, via pivotal genes including GPX4 and TFR1. METTL3-driven m6A modification on GPX4 mRNA sped up its decay via YTHDF2 recognition, thereby driving ferroptosis (Yang et al., 2023b). The current hypothesis holds that AAA disrupts RNA m6A modification in HK-2 cells, triggering downstream adverse biological effects that drive toxic cell injury.

As a representative m6A reader, IGF2BP2 stabilizes m6A-modified transcripts to mediate diverse cellular responses. Recent studies demonstrate that IGF2BP2 participates in the regulation of oxidative stress, autophagy and ferroptosis. It supports GPX4 expression to restrain lipid peroxidation and ferroptosis, maintains cellular redox balance, and dynamically controls autophagic activity in a context-dependent manner (Zhu et al., 2025; Yang et al., 2023a; Wang et al., 2024; Wu et al., 2025; Xu et al., 2022). Hence, exploring IGF2BP2 may provide new insights into the mechanisms of renal tubular cell toxicity.

## 2 Materials and methods

### 2.1 Chemical substances and cell cultivation

AAA was obtained from Must Bio-Technology (Chengdu, China). ^1^H and ^13^C NMR spectra were acquired on a 400 MHz Bruker AVANCE III spectrometer (Bruker BioSpin, Rheinstetten, Germany) to determine the structure. HPLC-based purity evaluation was conducted on a Dionex UltiMate 3,000 chromatographic system (Thermo Fisher Scientific, USA) equipped with a C18 column (4.6 mm × 250 mm, 5 µm). DMEM with 10% fetal calf serum (Gibco, New York, NY, USA) and 1% antimicrobial agents served as the growth medium for HK-2 cells (human renal proximal tubular epithelial cells), sourced from ATCC. Cultures were kept in a 37 °C, 5% CO_2_ environment. AAA was solubilized in DMSO, and the working DMSO concentration was 1% in cellular experiments.

### 2.2 Cell viability assay

HK-2 cells were dispensed into 96-well plates at 1 × 10^4^ cells per well and incubated overnight at 37 °C to allow attachment (Thermo Fisher, Langenselbold, Germany). Treatment with AAA, 0, 3.125, 6.25, 12.5, 25, 50, 100, and 200 μM, was carried out for 48 h. Each well received 10 μL of 5 mg/mL MTT reagent and was incubated for 4 h at 37 °C without light. After aspirating the supernatant, the formazan crystals were dissolved in 100 μL of DMSO. A multifunctional microplate reader (BioTek, Santa Clara, CA, USA) was employed to measure absorbance at 490 nm.

### 2.3 PharmMapper-based target prediction

From the PubChem database, the structure of AAA was retrieved and optimized via energy minimization, generating a mol2 file. After PharmMapper-based target prediction, UniProt IDs were unified and translated into gene symbols, enabling downstream enrichment analysis to reveal candidate targets (Wu et al., 2024).

### 2.4 RNA extraction of HK-2 cells

HK-2 cells were seeded in 6-well plates and incubated for 48 h with AAA at final concentrations of 0, 3.125, 12.5, and 50 μM. Total RNA was extracted using TRIzol reagent (Invitrogen, Carlsbad, CA, USA), and the resulting RNA concentration was quantified on a Qubit 3.0 fluorometer (Thermo Fisher, Waltham, MA, USA).

### 2.5 High-throughput sequencing

HK-2 cells were exposed to 12.5 μM AAA for 48 h, then total RNA was isolated. Seqhealth, based in Wuhan, China, performed the mRNA sequencing and MeRIP-based sequencing assays. Fragmented mRNAs (100–200 nt) were split into input and m6A-IP fractions. Both fractions were used to construct UMI-tagged libraries and subsequently sequenced on an Illumina NovaSeq 6,000 platform (San Diego, CA, USA) (Wu et al., 2022).

### 2.6 Bioinformatics analysis

Raw read quality was assessed and adapters were removed by Trim Galore. After adapter and quality trimming, clean reads were aligned to the hg19 reference genome using Hisat2. Transcript abundance was quantified by StringTie, while DEGs identification was performed with DESeq2 (Wu et al., 2022). Differential methylation analysis and peak calling of m6A marks were performed by ExomePeak2. STREME was then employed to discover m6A-associated sequence motifs (Meng et al., 2013; Bailey, 2021). RMDisease was utilized to evaluate the disease relevance of m6A methylation sites (Song et al., 2023). The distributions of m6A methylated sites were visualized through IGV (version 2.16). The mRNA-seq and MeRIP-seq datasets have been uploaded to the Gene Expression Omnibus (GEO) repository (ID:GSE328768).

### 2.7 Molecular docking

The interaction between AAA and the differentially expressed m6A regulators was explored through molecular docking using AutoDockTools v1.5.7. Experimentally determined structures for FTO (5DAB), YTHDF1 (4RCJ), IGF2BP2 (6ROL), and YTHDC2 (6K6U) were obtained from the PDB, whereas structural models for VIRMA (Q69YN4), RBM15B (Q8NDT2), ZC3H13 (Q5T200), and METTL5 (Q9NRN9) were retrieved from UniProt. All protein structures used for molecular docking are listed in Table 1. Then preprocessed with Ligplot and PYMOL. This preprocessing step involved the removal of surplus ligands, redundant peptide chains, and water molecules.

**TABLE 1:** The mRNA expression levels of m6A regulators in AAA-treated HK2 cells.

Gene	Regulation	Base mean	log_2_ fold change	*p* value	*p* adjust
*RBM15B*	Writer	2,140	−0.95	2.43 × 10^−32^	1.72 × 10^−30^
*IGF2BP2*	Reader	7,050	−0.52	1.75 × 10^−8^	1.82 × 10^−7^
*ZC3H13*	Writer	10,757	0.31	2.51 × 10^−6^	1.82 × 10^−5^
*YTHDF1*	Reader	1868	0.41	3.45 × 10^−6^	2.44 × 10^−5^
*YTHDC2*	Reader	1,083	0.31	2.03 × 10^−4^	9.84 × 10^−4^
*VIRMA*	Writer	3,839	0.27	3.06 × 10^−4^	1.42 × 10^−3^
*METTL5*	Writer	587	−0.49	8.69 × 10^−4^	3.60 × 10^−3^
*HNRNPA2B1*	Reader	68,340	−0.15	3.39 × 10^−3^	1.19 × 10^−2^
*FTO*	Eraser	1,269	0.34	1.15 × 10^−2^	3.42 × 10^−2^
*IGF2BP1*	Reader	2,864	−0.22	1.17 × 10^−1^	2.29 × 10^−1^
*WTAP*	Writer	4,116	−0.15	1.24 × 10^−1^	2.39 × 10^−1^
*HNRNPC*	Reader	15	−0.08	2.31 × 10^−1^	3.79 × 10^−1^
*IGF2BP3*	Reader	3,209	−0.06	2.82 × 10^−1^	4.40 × 10^−1^
*YTHDF2*	Reader	2042	0.07	3.01 × 10^−1^	4.61 × 10^−1^
*METTL3*	Writer	1,164	−0.07	3.73 × 10^−1^	5.39 × 10^−1^
*YTHDC1*	Reader	10,136	0.09	5.22 × 10^−1^	6.81 × 10^−1^
*CBLL1*	Writer	1,037	−0.05	5.24 × 10^−1^	6.83 × 10^−1^
*FMR1*	Reader	3,340	−0.03	5.93 × 10^−1^	7.41 × 10^−1^
*ALKBH5*	Eraser	2,487	0.03	6.50 × 10^−1^	7.85 × 10^−1^
*METTL14*	Writer	1,365	−0.04	6.65 × 10^−1^	7.96 × 10^−1^
*RBM15*	Writer	909	0.21	7.70 × 10^−1^	8.67 × 10^−1^
*YTHDF3*	Reader	1,446	−0.09	7.78 × 10^−1^	8.73 × 10^−1^

### 2.8 Measurement of intracellular ROS

Exposing cells to 0, 3.125, 12.5, and 50 µM AAA for 48 h, intracellular ROS levels were assessed. For co-treatment assays, cells were exposed to 5 mM NAC and 12.5 µM AAA for 48 h. Cells were incubated with the DCFH-DA probe, rinsed with PBS to remove non-internalized probe, and subsequently stained with Hoechst 33,342 for nuclei. Fluorescence micrographs were processed in ImageJ to quantify the intracellular ROS signal.

### 2.9 Measurement of intracellular Fe^2+^ levels

HK-2 cells were seeded 4 × 10^5^/well in 24-well plates were treated with AAA for 0 and 12.5 µM, 48 h. For Fer-1 combination, cells were pre-treated with 1 µM Fer-1 for 2 h followed by 12.5 µM AAA for 48 h, then stained sequentially with Hoechst 33,342 and FerroOrange, and imaged for intracellular Fe^2+^.

### 2.10 Measurement of intracellular lipid peroxidation

Cells were seeded and treated with AAA as described above; positive controls received LpoUp for 4 h. After treatment, cells were incubated with 2 µM Bodipy 581/591 C11 (37 °C, 30 min), stained with Hoechst 33,342, and imaged for lipid peroxidation in red/green ratio.

### 2.11 RT-qPCR analysis

The HK-2 cells were treated with 0, 3.125, 12.5, 50 µM AAA for 48 h. Total RNA was reverse transcribed to cDNA using a premix kit for reverse transcription (Accurate Biology, Changsha, China). Quantitative real-time PCR was performed with SYBR Green ProTaq HS Premix II on an AriaMX instrument (Agilent, Santa Clara, CA, USA). Relative mRNA levels were calculated by the 2^−ΔΔCT^ method, with GAPDH as the internal reference. Detailed primer sequences are provided in Table 2.

**TABLE 2:** Sequences of primers used in RT-qPCR.

Gene	Forward primer (5′–3′)	Reverse primer (5′–3′)
Beclin-1	ATC​TAA​GGA​GCT​GCC​GTT​ATA​C	CTC​CTC​AGA​GTT​AAA​CTG​GGT​T
*LC3*	GCC​TTC​TTC​CTG​CTG​GTG​AAC​C	TCC​TCG​TCT​TTC​TCC​TGC​TCG​TAG
*p62*	TGA​TTG​AGT​CCC​TCT​CCC​AGA​TGC	CCG​CTC​CGA​TGT​CAT​AGT​TCT​TGG
ACSL4	GGA​TTT​CCA​CAG​GGC​ATC​CA	GAT​CTG​ATG​GGA​CGG​GCT​TC
SLC7A11	TGT​GGG​AAT​GTC​ACT​GGT​GG	ACG​CCT​CCA​GAA​TGT​ACG​TG
GPX4	TCG​CTG​TCT​GCT​GTT​TCA​GTA	GTC​CTC​TAG​CTG​TGC​AGA​CA
GAPDH	TGA​CAT​CAA​GAA​GGT​GGT​GAA​GCA​G	GTG​TCG​CTG​TTG​AAG​TCA​GAG​GAG

### 2.12 Western blot analysis

HK-2 cells were challenged with 0, 3.125, 12.5, and 50 μM AAA for 48 h. For pharmacological intervention, cells were divided into separate treatment groups: co-treatment with 5 mM NAC and 12.5 µM AAA for 48 h, or pre-incubation with 1 µM Fer-1 for 2 h followed by 12.5 µM AAA for 48 h. Whole-cell lysates were prepared using RIPA lysis buffer supplemented with protease inhibitors and centrifuged at 12,000 × g for 15 min. The protein samples were fractionated on a 12% SDS-PAGE gel and electrotransferred to PVDF membranes. Blocking was done with 5% non-fat milk, followed by an overnight incubation at 4 °C with primary antibodies against LC3 (Selleck), p62 (Proteintech), Beclin 1 (Proteintech), GPX4 (Selleck), SLC7A11 (ABclonal), and ACSL4 (Proteintech). After extensive washing, the blots were incubated with HRP-conjugated secondary antibodies (Proteintech) for 90 min. Chemiluminescent bands were visualized on an Amersham Imager 680 system (Cytiva) and quantified densitometrically using ImageJ. Three independent biological replicates were performed.

### 2.13 Statistical analysis

Statistical comparisons were performed with one-way analysis of variance (ANOVA) using SPSS 26 (IBM). Data are shown as mean ± SD, and a *p* value less than 0.05 was taken to indicate statistical significance. Every experiment was repeated independently at least three times.

## 3 Result

### 3.1 Structure determination, purity evaluation, and predicted targets of AAA

^1^H and ^13^C spectra (Figures 1B,C) showed AAA key signals consistent with the formula C_17_H_11_NO_7_. HPLC-based purity analysis gave a single major peak at 15.49 min (Figure 1D). Peak area integration indicated a purity of 98.51%.

**FIGURE 1:**
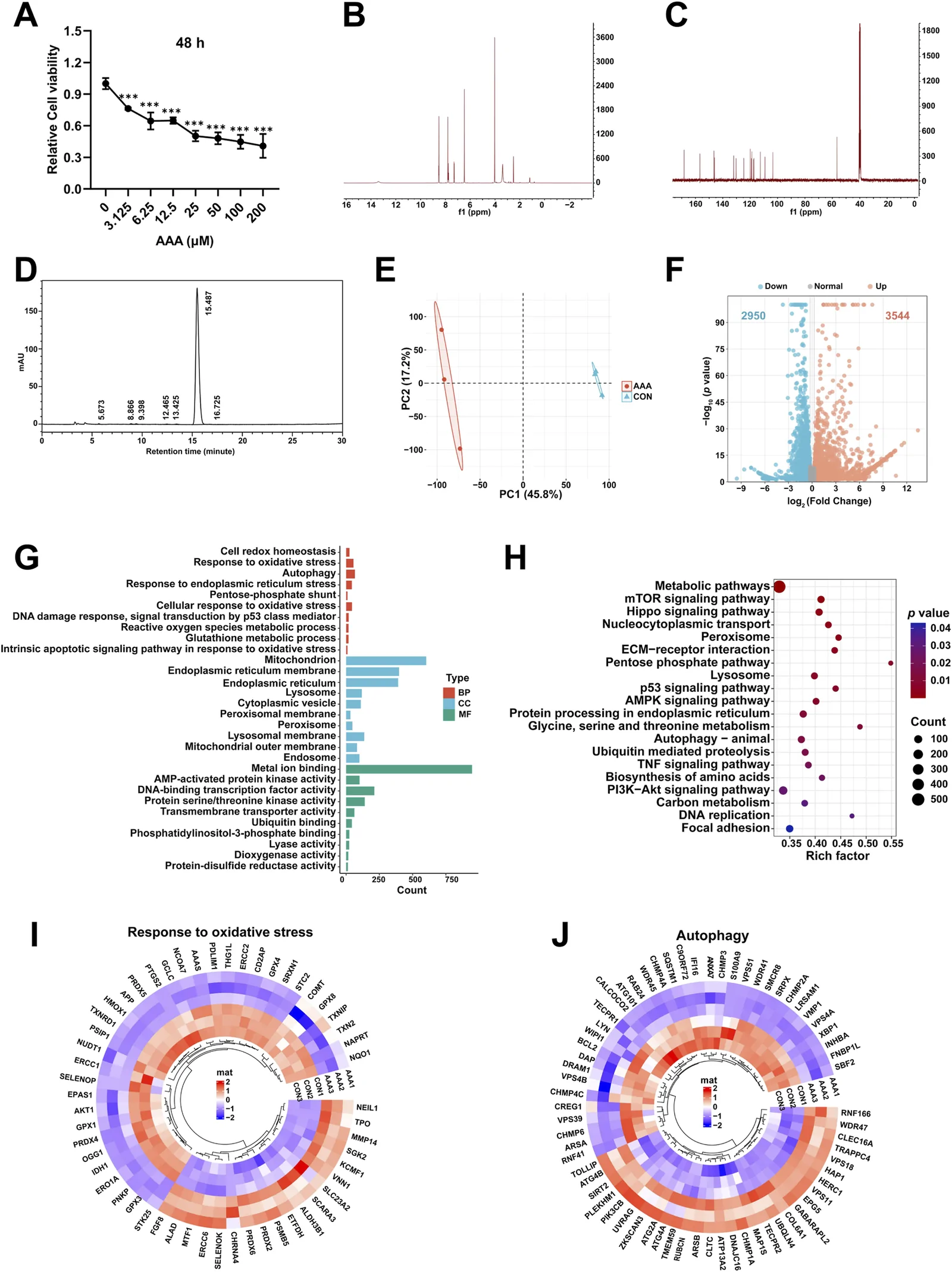
Basic characterization of AAA and mRNA-seq profiling of HK-2 cell line treated with 12.5 μM AAA for 48 h and corresponding controls. **(A)** MTT-based assessment of HK-2 cell viability after a 48 h challenge with AAA; ns, *p* > 0.05; *p < 0.05; **p < 0.01; ***p < 0.001; n = 3. **(B)** ^1^H and **(C)** ^13^C NMR spectra (400 MHz) of AAA. **(D)** HPLC purity assessment of AAA. **(E)** PCA plot of mRNA expression patterns between untreated and AAA-exposed groups. **(F)** A volcano diagram displaying differentially expressed genes (DEGs) after AAA treatment. **(G)** Enriched GO categories and **(H)** KEGG pathways identified from the DEGs. Circular heatmaps depicting the expression patterns of DEGs associated with **(I)** oxidative stress responses and **(J)** autophagy.

### 3.2 Dose- and time-dependent suppression of HK-2 cell viability by AAA

HK-2 cell viability was diminished by AAA in a manner that depended on both concentration and treatment duration. After 48 h treatment with 3.125, 12.5, and 50 μM, the viability decreased to 76.2%, 64.9%, and 48.0%, respectively (Figure 1A).

### 3.3 mRNA-seq profiling of differentially expressed genes (DEGs) in HK-2 cells treated with AAA

To investigate the molecular mechanisms through which AAA damages renal cells, mRNA-seq was performed on HK-2 cells after exposure to 12.5 μM of the compound for 48 h. Principal Component Analysis (PCA) showed a clear distinction between the treated group and controls (Figure 1E). Applying the criteria *p* < 0.05 and |log_2_ (fold change)| > 0.585, we detected 6,494 DEGs, among which 3,544 were upregulated and 2,950 downregulated (Figure 1F). Functional annotation of these DEGs was carried out by GO and KEGG enrichment analyses through the KOBAS database. Overrepresented GO terms primarily comprised response to oxidative stress, autophagy, glutathione metabolism, lysosome, and metal ion binding (Figure 1G), whereas enriched KEGG pathways notably included autophagy–animal, PI3K-Akt signaling, and carbon metabolism (Figure 1H). A circular heatmap displayed expression shifts in gene sets linked to autophagy and oxidative stress responses (Figures 1I,J). GSEA revealed that, relative to controls, the AAA-exposed group displayed pronounced activation of phosphatidylethanolamine metabolism, iron ion binding, and calcium ion transmembrane transport, while pathways such as regulation of cellular oxidative stress response, regulation of autophagosome assembly, and glutamine metabolism were suppressed (Figures 2A–F).

**FIGURE 2:**
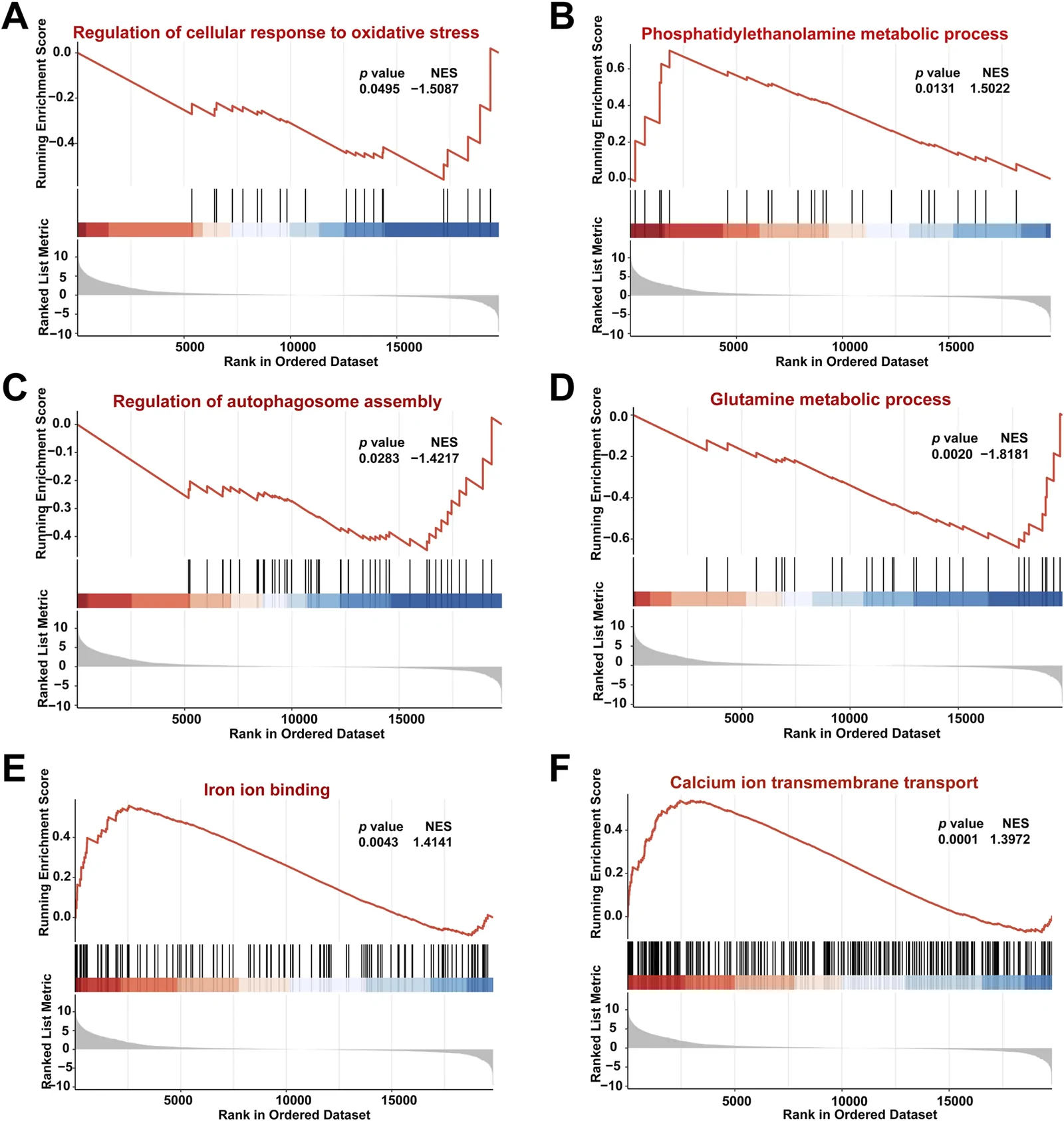
Investigation of critical biological processes and candidate regulatory pathways associated with AAA treatment. GSEA highlighted significant enrichment of pathways linked to **(A)** regulation of cellular response to oxidative stress, **(B)** phosphatidylethanolamine metabolic process, **(C)** regulation of autophagosome assembly, **(D)** glutamine metabolic process, **(E)** iron ion binding, and **(F)** calcium ion transmembrane transport.

### 3.4 m6A epitranscriptomic profiling of HK-2 cells upon AAA treatment

To assess whether AAA can reshape the m6A methylome of HK-2 cells, transcriptome-wide MeRIP-seq was carried out. A total of 42,708 m6A peaks spanning 10,065 genes were identified in the control group, while the AAA-treated group yielded 42,046 peaks across 9,984 modified genes (Figures 3A,B). The conserved DRACH motif (D = A/G/U, R = A/G, H = A/C/U) was strongly enriched in both groups according to STREME (Figure 3C). Over 6,000 transcripts per condition harbored one to three m6A peaks, whereas only a very small fraction carried more than five modification sites (Figure 3D). RMDisease analysis indicated that 45.8% of m6A-modified genes and 25.0% of m6A peaks are associated with disease (Figure 3E). Regarding the transcript-level distribution, m6A modifications were predominantly located in CDS regions, and ncRNA-associated modifications were concentrated in their corresponding ncRNA regions (Figures 3F,G).

**FIGURE 3:**
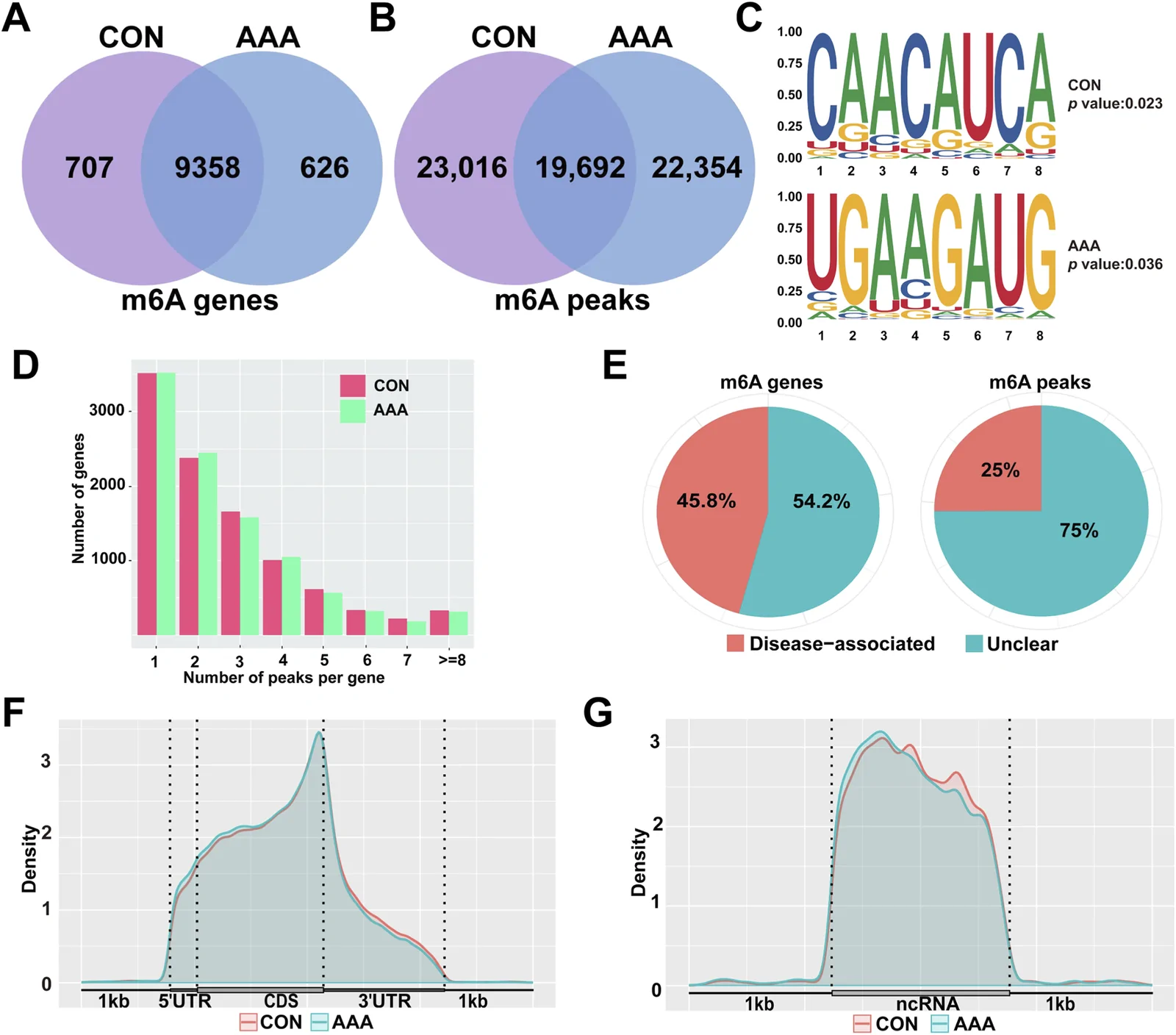
Profiling of m6A methylation patterns in HK-2 cells after AAA exposure. Venn diagrams display overlapped. **(A)** m6A-modified genes and **(B)** m6A peaks in control and AAA-treated groups. **(C)** Enriched sequence motifs of m6A peaks in both conditions. **(D)** Frequency distribution of m6A peak numbers per gene in control and AAA-treated cells. **(E)** Pie charts illustrating the fraction of disease-associated m6A genes and m6A peaks. Density distribution of m6A modifications along **(F)** mRNA and **(G)** ncRNA transcripts.

### 3.5 Conjoint investigation of mRNA-seq and MeRIP-seq datasets

Applying a significance threshold of *p* < 0.05, MeRIP-seq identified 1,874 mRNAs carrying differential m6A modification, while mRNA-seq yielded 7,508 DEGs between the AAA-treated group and controls. The intersection of these two datasets contained 1,006 overlapping genes (Figure 4A). When an additional filter of |log_2_ (fold change)| > 0.263 was imposed, the DEG count dropped to 727, consisting of 271 up- and 456 downregulated transcripts (Figure 4B). Functional enrichment was subsequently performed through KOBAS. GO analysis indicated that the gene set was strongly linked to cellular oxidative stress response, autophagy and its regulation, response to oxidative stress, and glutathione metabolism; correspondingly, KEGG enrichment highlighted mitophagy, autophagy, and ferroptosis (Figures 4C,D). GSEA further identified differentially enriched pathways when comparing the AAA-treated cohort with controls (Figures 4E–H).

**FIGURE 4:**
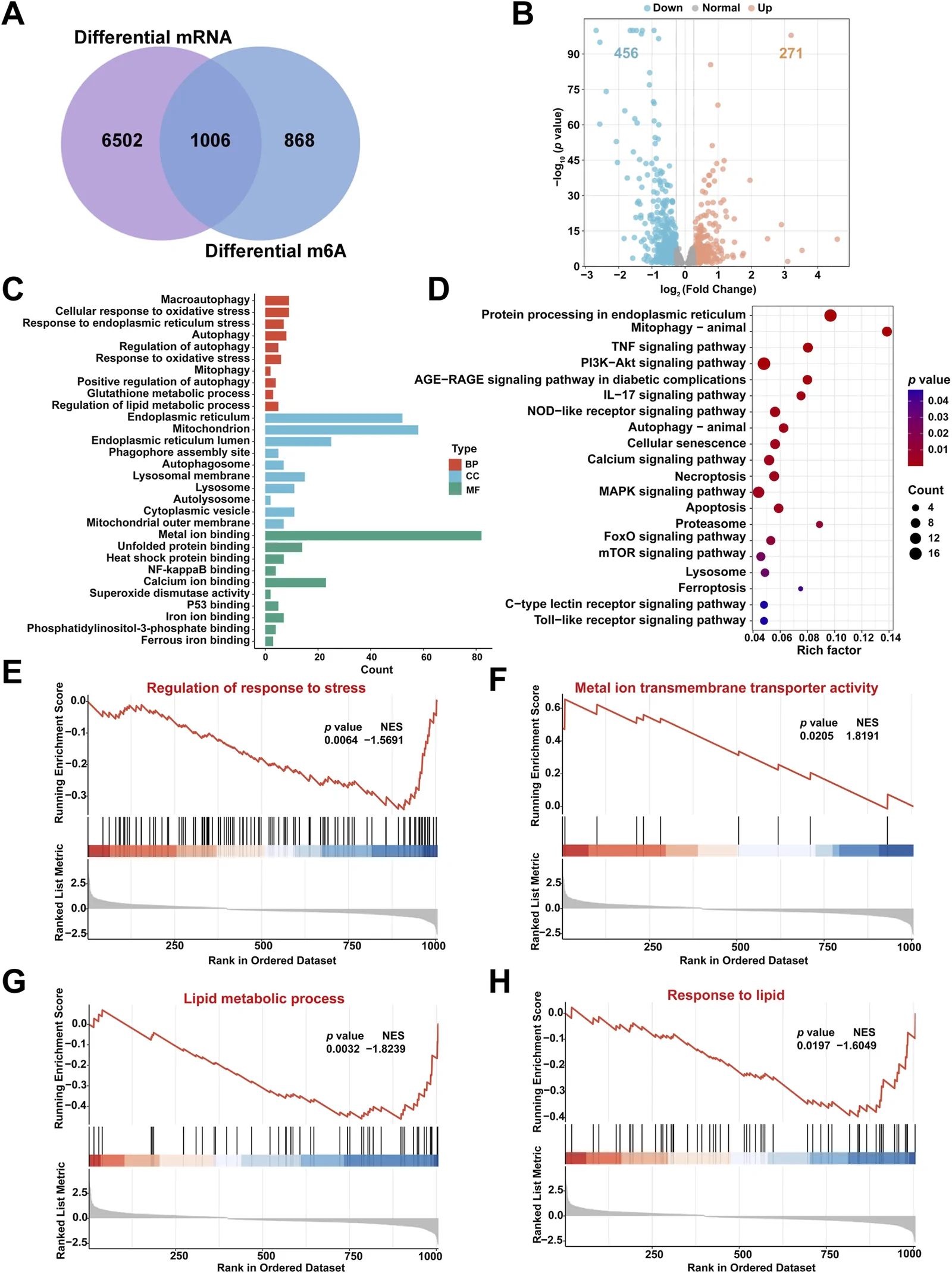
mRNA expression and m6A modification alterations in AAA-treated HK-2 cells. **(A)** Venn diagram depicting shared genes between DEGs and m6A-modified transcripts. **(B)** Volcano plot of differentially expressed genes. Representative **(C)** GO biological categories and **(D)** KEGG-enriched pathways for genes responsive to AAA. GSEA identified significant enrichment of **(E)** regulation of response to stress, **(F)** metal ion transmembrane transporter activity, **(G)** lipid metabolic process, and **(H)** response to lipid.

### 3.6 Interaction study of AAA with altered m6A modification regulators

The mRNA-seq and MeRIP-seq datasets were utilized to profile m6A modification patterns and quantify the expression of m6A regulatory factors (Table 3). The expression of ZC3H13, YTHDF1, YTHDC2, VIRMA, and FTO was substantially increased, while that of RBM15B, IGF2BP2, and METTL5 was clearly reduced.

**TABLE 3:** The interactions between AAA and m6A regulators.

Protein	PDB/Uniprot ID	Binding energy (KJ/mol)	H-bond number	Residues involved in H-bond formation	Hydrophobic contact number	Residues involved in hydrophobic contacts
FTO	5DAB	−7.85	7	Leu51 (3 H-Bonds), Arg52 (3 H-Bonds), Lys160	6	Tyr39, Trp42, Gln43, Pro47, Leu49, Ile50
YTHDF1	4RCJ	−7.43	5	Met428, Ser429, Val434, Lys453 (2 H-Bonds)	6	Phe425, Arg426, Ser430, Gly432, Pro433, Glu451,
IGF2BP2	6ROL	−7.39	2	Asn431,His513	8	Glu47, Phe433, Pro465, Ala466, Glu467, Val471, Arg474, Met475,
VIRMA	Q69YN4	−7.03	1	Pro334	6	Leu330, Val333, Pro335, Met336, Phe804, Phe1706
YTHDC2	6K6U	−6.74	7	Gly221, Tyr319 (3 H-Bonds), Ser1304, Gln1305, Gly1308, Val1371, Trp1373 (2 H-Bonds), Lys1376	4	Gln1306, Lys1307, Lys1370, Glu1372
RBM15B	Q8NDT2	−6.36	9	Arg220 (4 H-Bonds), Val322	4	Tyr218, Leu219, Pro320, Ala321
ZC3H13	Q5T200	−5.18	4	Asp1326,Arg1335 (3 H-Bonds)	3	Trp1323,Ala1327,Trp1331
METTL5	Q9NRN9	−5.05	4	Ala94, Glu98, Leu99 (2 H-Bonds)	5	Asn91, Glu95, Thr100, Ile102, Met104

Molecular docking simulations between AAA and these differentially regulated m6A regulators were carried out to find potential targets of AAA. Binding free energies were estimated with AutoDock, accounting for van der Waals interactions, electrostatics, hydrogen bonds, desolvation, intramolecular energies, and torsional contributions. The resulting docking models and energy values for AAA in complex with FTO, YTHDF1, IGF2BP2, VIRMA, YTHDC2, RBM15B, ZC3H13, and METTL5 (Figure 5; Table 3). Binding energies more negative than −5 kcal/mol were interpreted as stable binding between AAA and these m6A regulators. Combining the mRNA-seq findings with molecular docking data identified IGF2BP2 as a likely molecular target of AAA.

**FIGURE 5:**
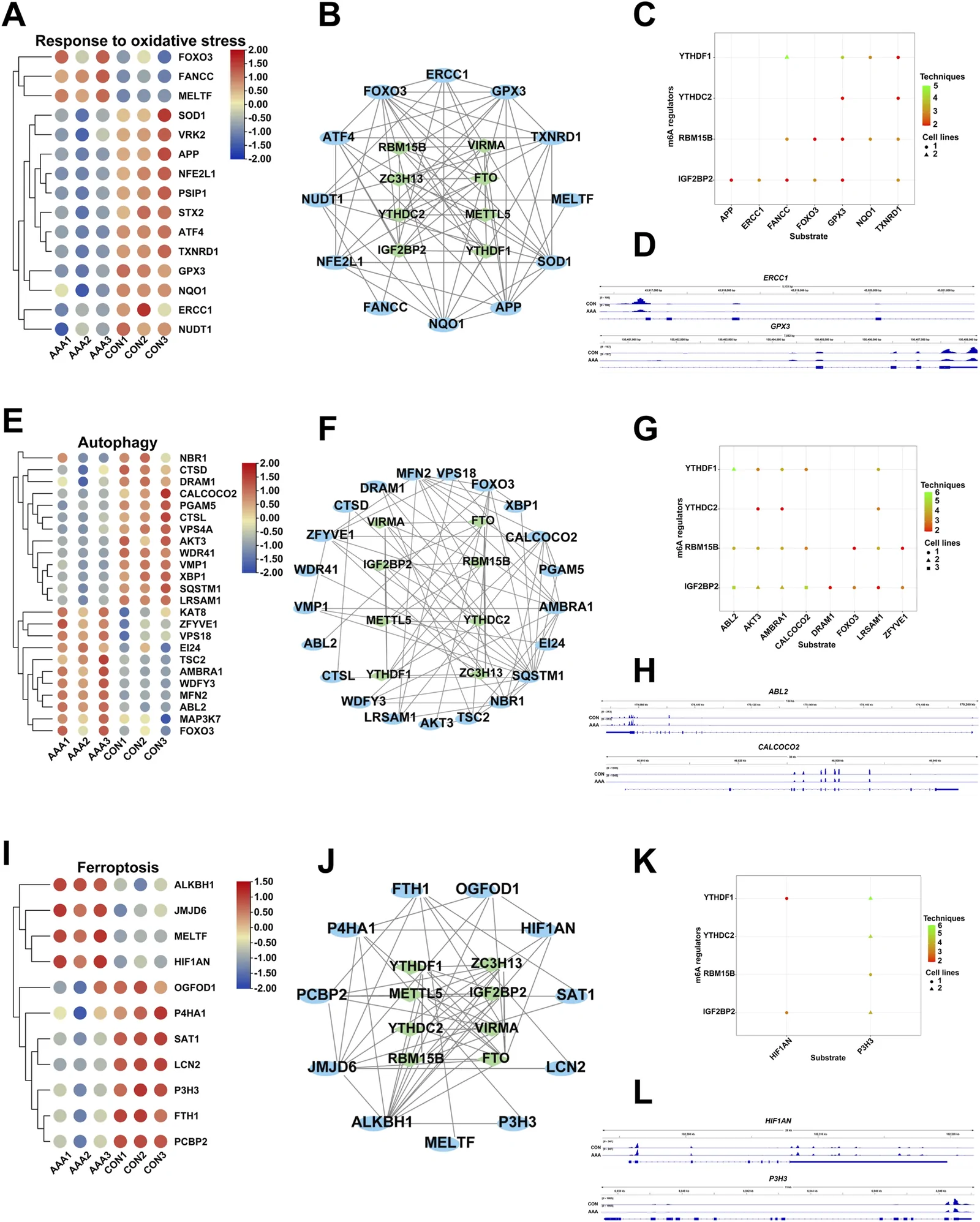
Putative m6A regulators showing differential expression in response to AAA treatment. Rectangular heatmaps display expression profiles of **(A)** response to oxidative stress. Interaction networks connecting m6A regulators to **(B)** response to oxidative stress-related genes. Predicted substrates of the m6A regulators based on gene sets involved in **(C)** response to oxidative stress-related pathways. IGV views of m6A modification levels on mRNA transcripts of **(D)** ERCC1 and GPX3 of the response to oxidative stress-related genes. **(E–H)** The Rectangular heatmaps, PPI, predicted substrates of autophagy and IGV views of ABL2 and CALCOCO2 of autophagy-related genes. **(I–L)** The Rectangular heatmaps, PPI, predicted substrates of ferroptosis and IGV views of HIF1AN and P3H3 of ferroptosis -related genes.

### 3.7 Interplay between altered m6A regulators and genes linked to oxidative stress, autophagy, and ferroptosis

Functional annotation using GO and KEGG resources highlighted a significant enrichment of pathways connected to oxidative stress, autophagy, and ferroptosis. Changes in the expression of genes within these three categories were further visualized through rectangular heatmaps (Figures 6A,E,I). The STRING platform was used to generate protein–protein interaction (PPI) networks, enabling an exploration of potential regulatory crosstalk between dysregulated m6A regulators and their target genes within the corresponding pathways. In the oxidative stress gene set, SOD1, GPX3, and FOXO3 each recorded a degree of 5, NQO1 had 4, and both APP and TXNRD1 had a degree of 3. Among autophagy-related factors, SQSTM1 attained the highest degree (15), followed by AMBRA1 (10), MFN2 (9), CALCOCO2 (8), and FOXO3, NBR1, and ZFYVE1, each with a degree of 7. For genes tied to ferroptosis, ALKBH1 showed a degree of 13, JMJD6 had 9, P4HA1, FTH1, and PCBP2 all exhibited 6, while HIF1AN and OGFOD1 had a degree of 5 (Figures 6B,F,J). Analysis was applied to identify potential RNA substrates of m6A methylation factors. Four regulators, YTHDF1, YTHDC2, RBM15B, and IGF2BP2, were selected to assess their links with oxidative stress-, autophagy-, and ferroptosis-associated genes (Figures 6C,G,K). Visualization with the Integrative Genomics Viewer (IGV) revealed elevated m6A modification levels in the AAA-treated group on the oxidative stress genes ERCC1 and GPX3, the autophagy genes ABL2 and CALCOCO2, and the ferroptosis genes HIF1AN and P3H3 (Figures 6D,H,L).

**FIGURE 6:**
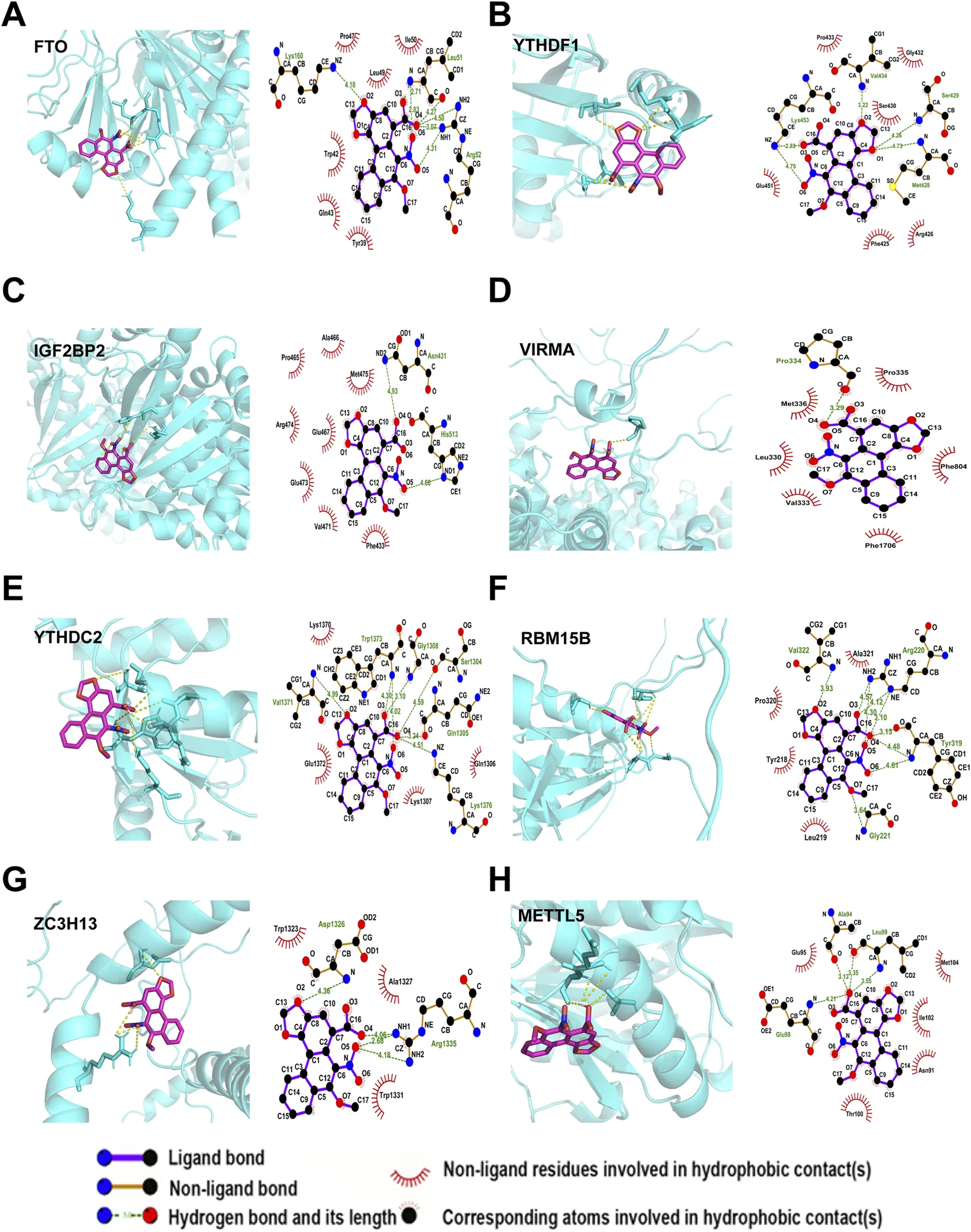
AAA binding to core m6A regulators. 3D and 2D docking views of AAA complexed with **(A)** ZC3H13, **(B)** YTHDF1, **(C)** YTHDC2, (D) VIRMA, **(E)** FTO, **(F)** RBM15B, **(G)** IGF2BP2, and **(H)** METTL5.

### 3.8 Stable binding of AAA to core m6A modification regulators

To confirm direct interactions between AAA and writer components, AutoDock was used to dock AAA individually with eight central m6A regulators: FTO, YTHDF1, IGF2BP2, VIRMA, YTHDC2, RBM15B, ZC3H13, and METTL5. Basic protein properties, docking binding energies, numbers of hydrogen bonds, and the amino acid residues forming these hydrogen bonds are listed, together with 2D and 3D diagrams of each AAA-protein complex (Figures 5A–H; Table 3). The calculated binding energies of AAA toward FTO, YTHDF1, IGF2BP2, VIRMA, YTHDC2, RBM15B, ZC3H13, and METTL5 were −7.85, −7.43, −7.39, −7.03, −6.74, −6.36, and −5.18 kJ/mol, respectively. All values fell below −5 kJ/mol, pointing to stable complex formation between AAA and these eight regulatory factors. This observation fits with previously described mechanisms in which small molecules bind tightly to target proteins via hydrogen-bonding and thereby modulate their activity. The molecular docking results suggest predict potential binding between AAA and its target, offering a preliminary structural hint to decipher the regulatory effect of AAA on m6A methylation.

### 3.9 Response to oxidative stress induced by AAA

In AAA-treated cells, intracellular reactive oxygen species (ROS) levels rose in a concentration-dependent manner, with fluorescence intensity increasing markedly. This disrupted the cellular redox balance and triggered oxidative stress (Figures 7A,B). In the rescue experiments, ROS fluorescence values were 2.09 for the control, 7.84 for AAA treatment, 2.11 for AAA and NAC co-treatment, and 5.04 for the positive control. Both AAA and positive control groups showed significantly higher ROS levels than the control and AAA and NAC groups, whereas no significant difference was observed between the latter two (Figures 7J,K).

**FIGURE 7:**
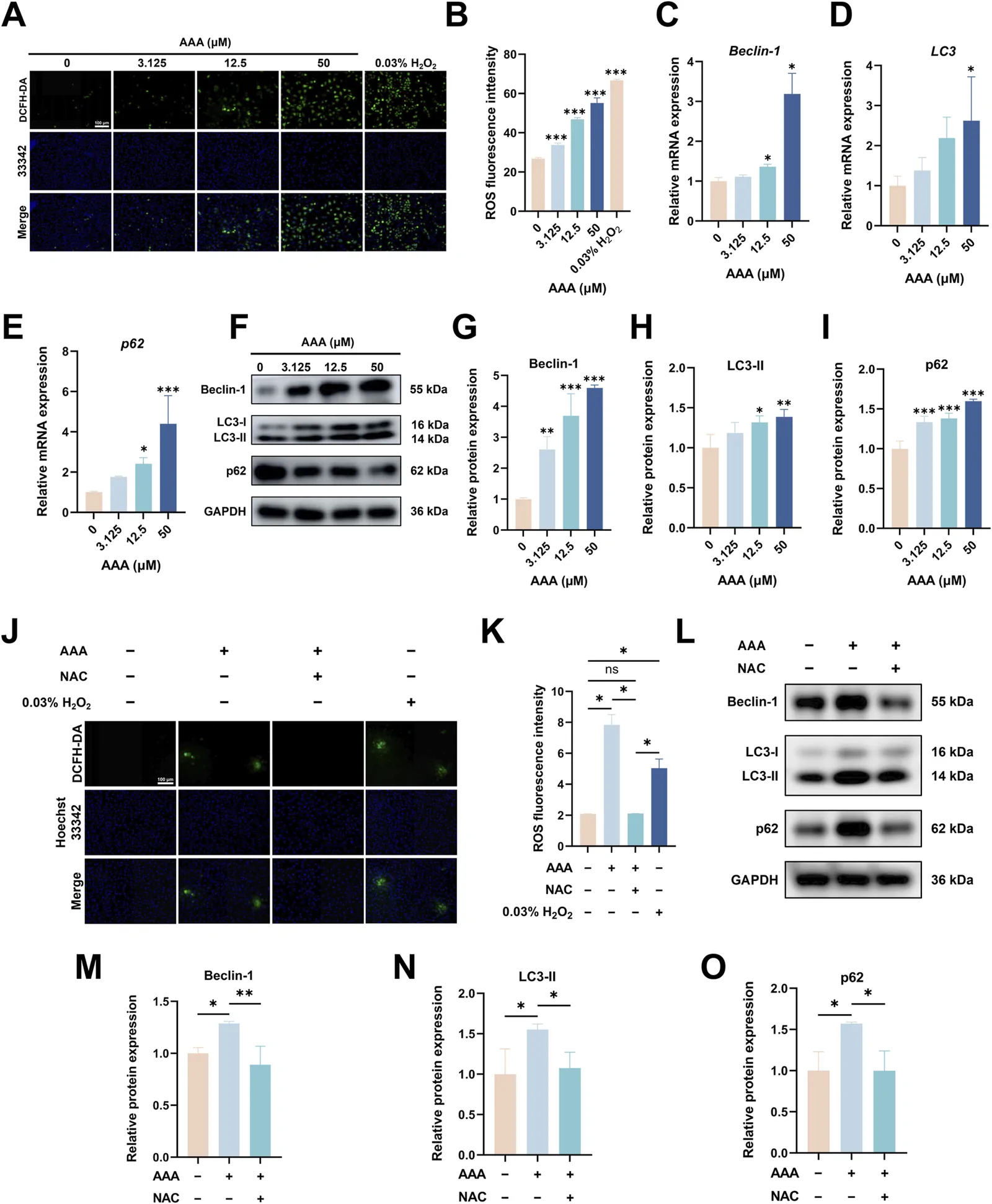
AAA-triggered oxidative stress and autophagy responses in HK-2 cells after 48 h exposure. **(A)** Representative ROS fluorescence micrographs and **(B)** corresponding quantification of fluorescence intensity. Autophagy-related mRNA levels of **(C)** Beclin-1, **(D)** LC3-II, and **(E)** p62 assessed by RT-qPCR. **(F)** Immunoblot analysis of Beclin-1, LC3-II, and p62 protein expression in AAA-treated HK-2 cells. Semiquantitative densitometry values for **(G)** Beclin-1, **(H)** LC3-II, and **(I)** p62. AAA induces ROS production and autophagy in HK-2 cells, and NAC reverses these effects.**(J)** Intracellular ROS levels detected in group 0, 12.5 μM AAA, 12.5 μM AAA and 5 mM NAC, 0.03% H_2_O_2_. **(K)** Quantification of ROS fluorescence intensity. (L–O) Western blot analysis of autophagy related protein expression and the corresponding quantitative analysis. Scale bar: 100 μm. ns, *p* > 0.05; *p < 0.05; **p < 0.01; ***p < 0.001; n = 3.

### 3.10 Autophagy in HK-2 cells induced by AAA

The upregulated expression of Beclin-1 and LC3-II in WB and qPCR suggested that autophagy initiation was activated and autophagy generation increased. At the same time, the increase of p62 expression indicates that the degradation link in the later stage of autophagy is blocked and the autophagy flow is not smooth, which shows an incomplete autophagy state with enhanced autophagy initiation but blocked autophagy flow (Figures 7C–I).

12.5 µM AAA significantly upregulated Beclin-1, LC3-II, and p62 to 1.29-, 1.57-, and 1.55-fold of the control levels, while co-treatment with 5 mM NAC reversed these increases, reducing the three proteins by 31%, 36%, and 30%, respectively, to near control levels in rescue assays (Figures 7L–O).

### 3.11 Ferroptosis in HK-2 cells induced by AAA

In the treatment group, as the crucial negative regulator of ferroptosis, the expression of the SLC7A11 and GPX4 was markedly downregulated, whereas the protein level of the pro-ferroptotic factor ACSL4 was significantly upregulated. These findings indicate that cystine uptake and glutathione biosynthesis are suppressed, the GPX4-mediated antioxidant defense system is impaired, and lipid peroxidation-related anabolism is potentiated. Collectively, these alterations ultimately trigger ferroptosis activation (Figures 8A–G).

**FIGURE 8:**
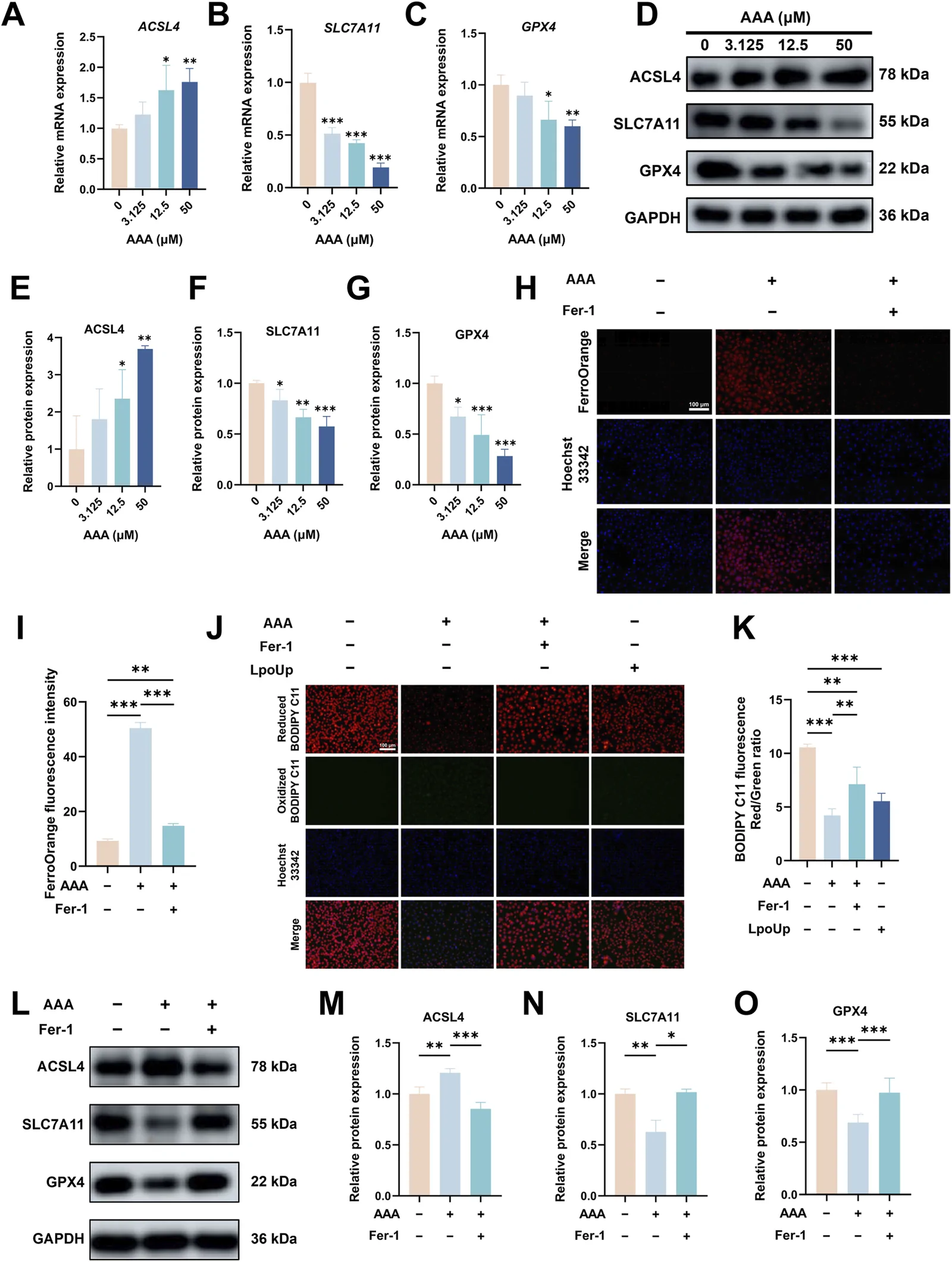
Effect of AAA on ferroptosis markers in HK-2 cells after 48 h exposure. RT-qPCR analysis of mRNA expression for ferroptosis-related genes **(A)** ACSL4, **(B)** SLC7A11, and **(C)** GPX4. **(D)** Protein expression of ACSL4, SLC7A11, and GPX4 was examined in AAA-treated HK-2 cells. Semi-quantitative densitometry results for **(E)** ACSL4, **(F)** SLC7A11, and **(G)** GPX4. Effects of Fer-1 intervention on AAA-induced ferroptosis-related changes in HK-2 cells. **(H)** Intracellular Fe^2+^ levels detected by FerroOrange fluorescence staining and **(I)** the corresponding quantitative analysis. **(J)** Lipid peroxidation detected by BODIPY 581/591 C11 staining and **(K)** analysis of the red/green fluorescence intensity ratio. (L–O) Western blot analysis of ferroptosis related protein expression and the corresponding quantitative analysis. Scale bar: 100 μm, ns, *p* > 0.05; *p < 0.05; **p < 0.01; ***p < 0.001; n = 3.

BODIPY 581/591 C11 and FerroOrange probes were used to detect lipid peroxidation and Fe^2+^ in HK-2 cells. Treatment with 12.5 µM AAA shifted BODIPY fluorescence to green and increased FerroOrange intensity, indicating elevated peroxidation and ferrous iron. Pre-treatment with 1 µM Fer-1 reversed both signals, restoring the red/green ratio and lowering FerroOrange to near-control levels (Figures 8H–K).

AAA upregulated ACSL4 by 1.21-fold and reduced SLC7A11 and GPX4 by 37% and 31%, respectively. Fer-1 pre-treatment reversed these changes, decreasing ACSL4 by 30% and raising SLC7A11 and GPX4 to 1.62- and 1.42-fold of control in the rescue experiments (Figures 8L–O).

## 4 Discussion

AAA is a kind of nitrophenanthrene organic acid in aristolochiaceae plants, exhibits remarkable pharmacological and toxicological properties. From the perspective of pharmacological toxicity, it has clear renal toxicity and carcinogenicity. Long-term or excessive exposure will cause renal tubulointerstitial fibrosis, which will lead to irreversible renal function damage and even induce malignant tumors in urinary system (Wang et al., 2025). At the application level, plants containing AAA have been used in traditional Chinese herbal medicines, but due to their serious toxicity risks, the use of related medicinal materials has been restricted in many countries (Hu et al., 2025; Leung and Chan, 2015). RNA-seq and MeRIP-seq were applied to HK-2 cells after AAA exposure to profile changes in m6A modification and mRNA expression. This cellular system was further used to clarify the toxicological actions of AAA through the lenses of oxidative stress, autophagy, and ferroptosis.

The m6A modification can affect oxidative stress levels by regulating mRNA stability or translation efficiency of genes related to antioxidant pathways. For example, m6A reader protein YTHDF1 can recognize and stabilize the mRNA of Nrf2, promote its translation and enhance cellular antioxidant capacity (Yang et al., 2025; Liu et al., 2022). On the contrary, if METTL3 of m6A writer is knocked down, it will reduce the m6A modification level of antioxidant genes, accelerate their mRNA degradation, lead to ROS accumulation and aggravate oxidative damage (Fu et al., 2025; Zhang et al., 2024). GSEA reveals a transcriptional profile marked by reduced representation of antioxidant defense genes among oxidative stress-related pathways. This expression pattern would compromise the cellular capacity for ROS elimination. Consequently, the impaired clearance of ROS would favor their intracellular accumulation, a state that aligns with the observed elevation in ROS levels at the functional level. The downregulation of lipid metabolism genes at the transcriptomic level reflects a disturbance in lipid homeostasis. Such dysregulation alters membrane lipid composition, increasing the abundance of peroxidation-susceptible lipid species. Meanwhile, impaired antioxidant defenses compromise ROS clearance. These changes collectively create a microenvironment conducive to ferroptosis, which is consistent with the elevated ROS fluorescence and positive BODIPY staining observed in experiments (Tang et al., 2026; Waldeck-Weiermair et al., 2026; Shi et al., 2025).

At the same time, m6A modification regulates the autophagy process by targeting genes related to autophagy. METTL3 can mediate m6A modification of mRNA of autophagy initiation genes such as LC3-II and Beclin-1, and promote their translation and initiate autophagy after being recognized by YTHDF2 (Li et al., 2023b; Zhang et al., 2025). On the other hand, m6A eraser FTO can remove m6A modification of ATG5 and ATG7, prolong their mRNA half-life and enhance autophagy flux (Hao et al., 2025). m6A indirectly modulates autophagy activation and suppression by controlling the expression of AMPK/mTOR pathway-related genes (Abouelhamd et al., 2025). GSEA enrichment analysis showed that autophagy-related pathways were inhibited as a whole, suggesting that the expression of autophagy-related genes was reduced at the transcriptional level. However, western blot results showed that p62 accumulation and LC3-II increased, indicating that autophagic flux was blocked and the fusion or degradation process between autophagy and lysosome was inhibited (Niu et al., 2026; Zhou et al., 2026). The data indicated that autophagic initiation was suppressed and late-stage autophagic degradation was markedly blocked, leading to the accumulation of p62 and LC3-II and manifesting as an impaired autophagic flux (Stevenson et al., 2026; Coronado-Monroy et al., 2026).

m6A modifies key molecules that target the ferroptosis core pathway to achieve regulation. For example, METTL3 can mediate m6A modification of GPX4 mRNA, and YTHDF2 will accelerate its degradation after recognition, reduce the level of GPX4 protein, and promote lipid peroxidation and ferroptosis (Qin et al., 2025). m6A regulates the expression of iron metabolism genes including SLC7A11 and ACSL4, disrupts intracellular iron homeostasis, and governs the onset of ferroptosis (Tian et al., 2026). HK-2 cells undergo ferroptosis, which is characterized by regulated change of SLC7A11, GPX4 and ACSL4 (Liu et al., 2025). Ranscriptomic changes in lipid metabolism genes indicate a disturbance in lipid homeostasis, which alters membrane lipid composition and renders cells more vulnerable to oxidative damage. This is corroborated by the protein and mRNA expression data, showing that ACSL4 is upregulated, whereas SLC7A11 and GPX4 are downregulated. Such molecular shifts favour the accumulation of peroxidizable lipids and compromise ROS clearance, consistent with the elevated ROS fluorescence and positive BODIPY staining. Importantly, the rescue effects of the antioxidant NAC and the ferroptosis inhibitor Fer-1 confirm the ferroptosis-specific nature of these phenotypes. Taken together, these results demonstrate that ferroptosis arises from lipid dyshomeostasis and impaired antioxidant defence, rather than being a mere consequence of transcriptional activation (Liu et al., 2025).

Under oxidative stress, cells try to inhibit excessive stress and cell death pathways at the transcriptional level, but since the stress intensity is beyond the compensatory range of transcriptional regulation, cells eventually activate autophagy and ferroptosis at the functional level through post-transcriptional regulation, protein stability changes and metabolic reprogramming (Wang et al., 2026; Alharbi et al., 2026). Enhanced autophagy may be involved in stress clearance as an early protective mechanism, but continued elevated ROS further inhibits SLC7A11-GPX4 antioxidant system and promotes lipid peroxidation accumulation, eventually breaking through the protective effect of autophagy and inducing ferroptosis (Ren et al., 2026; Liu et al., 2026).

As a canonical m6A reader, IGF2BP2 mediates post-transcriptional regulation by binding and stabilizing m6A-modified mRNA to control cellular stress responses. It critically regulates oxidative stress, autophagy and ferroptosis and enhances ferroptosis resistance. Mechanistically, IGF2BP2 sustains GPX4 expression by stabilizing its mRNA to eliminate lipid peroxides. IGF2BP2 dysfunction reduces GPX4 levels, promotes lipid peroxide accumulation and sensitizes cells to ferroptosis (Zhu et al., 2025; Yang et al., 2023a). It also maintains cellular redox homeostasis, and IGF2BP2 signaling disruption exacerbates oxidative stress and epithelial injury. IGF2BP2 modulates autophagic flux in a context-dependent manner. In renal epithelial cells, SIK2 supports normal autophagy turnover, while IGF2BP2 binds RNA transcripts to inhibit autophagy under specific conditions, thereby shaping autophagic status and cell fate (Wu et al., 2025; Xu et al., 2022). Collectively, IGF2BP2 functions as a central hub linking oxidative stress, autophagy and ferroptosis, and its investigation advances the mechanistic understanding of renal tubular injury.

Integrated molecular docking, substrate prediction and transcriptomic screening identified IGF2BP2 as a key candidate target. Although AAA treatment markedly altered RBM15B expression, RBM15B only showed moderate AAA-binding affinity and weak correlations with the three stress pathways. In comparison, IGF2BP2 displayed stronger AAA binding and extensive associations with stress signaling. The potential involvement of RBM15B in AAA-induced HK-2 cell injury still needs further validation.

## 5 Conclusion

The m6A modification landscape of mRNAs in AAA-treated HK-2 cells was characterized. Dysregulated m6A regulators and aberrant methylation of the IGF2BP2 transcript impaired mRNA stability, translation, storage, and decay (Figure 9). Thus, the current study points to a possible mechanism underlying AAA-induced nephrotoxicity in HK-2 cells. The regulation of oxidative stress, autophagy, and ferroptosis here is not solely determined at the transcriptional level. Instead, a stress-autophagy-ferroptosis cascade is coordinated through multiple layers of post-transcriptional control, which ultimately shapes cell fate.

**FIGURE 9:**
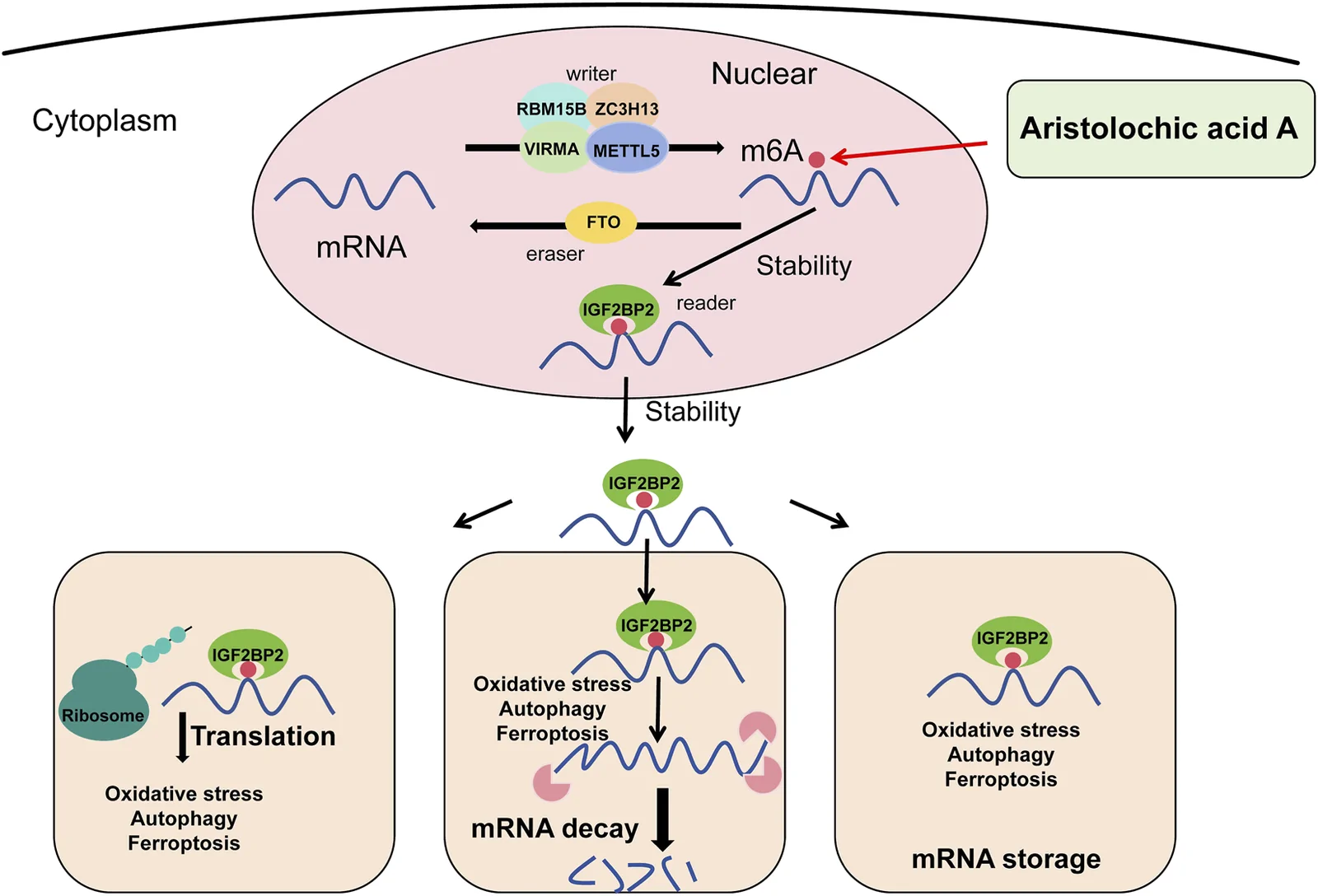
AAA induced dysregulated m6A methylation on genes involved in oxidative stress, autophagy, and ferroptosis, which consequently compromised the translation, storage, and decay of their mRNAs.

## Data Availability

The mRNA-seq and MeRIP-seq data generated in this study have been deposited in the NCBI Gene Expression Omnibus (GEO, https://www.ncbi.nlm.nih.gov/geo/, accessed on 22 April 2026) under accession numbers GSE328768 for the experimental group and GSE295178 for the control group. The original contributions presented in the study are included in the article/Supplementary Material, further inquiries can be directed to the corresponding authors.
